# Subarray programmable terahertz metasurface for optical logic and high-order amplitude modulation

**DOI:** 10.1038/s41377-026-02255-z

**Published:** 2026-05-07

**Authors:** Lan Wang, Sen Gong, Chaoming Xia, Dongyang Liu, Xuan Cong, Ao Zhu, Hongxin Zeng, Feng Lan, Ziqiang Yang, Taiichi Otsuji, Yaxin Zhang

**Affiliations:** 1 School of Physics, University of Electronic Science and Technology of China, Chengdu, 611731 China; 2 Engineering Center of Integrated Optoelectronic & Radio Meta-chips, Chengdu, 611731 China; 3 School of Electronic Science and Engineering, University of Electronic Science and Technology of China, Chengdu, 611731 China; 4 ENSEMBLE3 Center of Excellence Ltd., Warszawa, 01919 Poland; 5 International Research Institute of Disaster Science, Sendai, 9808572 Japan

**Keywords:** Terahertz optics, Sub-wavelength optics, Metamaterials

## Abstract

Integrated terahertz (THz) communication-sensing-computing systems require reconfigurable platforms that can simultaneously support logic operations and signal modulation. Here, we propose a subarray programmable THz metasurface that elevates the subarray to the minimum addressable unit. Within each subarray, a high electron mobility transistor (HEMT) serves as the active material; collective resonance tuning of the two-dimensional electron gas (2DEG) enables broadband control of array-level resonances and transmission amplitude. Using spatial domain combinatorial coding across subarrays, the device maps Boolean logic over a wide bandwidth and directly realizes four-level pulse amplitude modulation (PAM-4) at the wavefront. In a 220 GHz quasi-optical link, dynamic measurements demonstrate real-time Boolean functions up to 200 MHz and stable PAM-4, while a single-tone modulation reaches 6 GHz. This subarray-level gating strategy integrates optical logic and high-order amplitude modulation on the same hardware without increasing addressing complexity, providing a scalable route to compact, reconfigurable THz front ends for future communications and intelligent sensing.

## Abstract (web summary)

Subarray programmable THz metasurface using high-speed GaN HEMTs enables broadband control, 100–200 MHz optical logic, and PAM-4 modulation, delivering scalable reconfigurable THz front ends without added addressing complexity.

## Graphical abstract

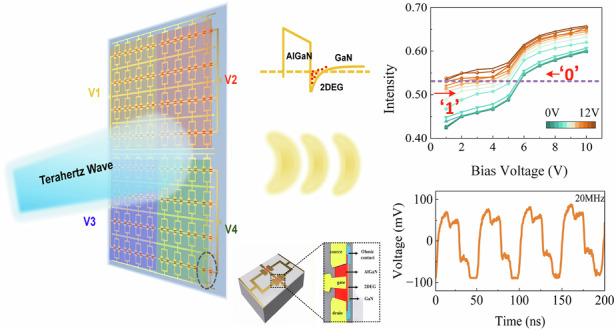

## Introduction

Sixth-generation (6 G) and beyond mobile systems are evolving from the single objective of transmitting bits towards a paradigm of integrated sensing, communication, and computing (ISCC)^1,2^. The terahertz band, characterized by its ultra-wide bandwidth and fine spatial resolution, is regarded as a key spectral region for this paradigm. In latency critical applications such as intelligent edge sensing for autonomous systems^3^, immediate event evaluation is paramount. This necessitates a seamless orchestration of sensing triggers, physical layer decision-making, and communication transmissions. Consequently, the underlying hardware platforms need to support coexistence of these functions, rather than just high-speed communication. Realizing such capabilities in the THz regime requires modulators that provide flexible, reconfigurable control of THz waves tailored to scenario-specific demands^4–6^. Yet the limited intrinsic response and restricted tunability of natural materials in this band leave THz control devices facing persistent bottlenecks.

Programmable metasurfaces provide a platform toward this vision and show potential for physical layer signal enhancement and indoor WLAN applications^7–10^, by discretely controlling subwavelength elements, they enable real-time manipulation of the electromagnetic waves in amplitude, phase, and polarization. With the introduction of dynamic mechanisms such as MEMS^11,12^, phase change materials^13,14^, and semiconductor active devices (e.g., diodes, HEMTs, graphene)^15–17^, research has progressed from static beam shaping to space-time coding and real-time multiplexing. These advances are continually expanding capability limits, positioning metasurfaces not just as transmitters, but as intelligent front-end processors capable of performing tasks ranging from holographic imaging to native physical encryption. Recent demonstrations in digital coding metasurfaces image processing further validate the system-level potential for ISCC^18,19^, suggesting a pathway where intelligent surfaces act as first-layer decision makers to enable real-time target recognition and secure data transmission.

Current THz programmable metasurfaces predominantly fall into two architectural classes. The first is pixel-level addressing, implemented on material such as Si-CMOS and two-dimensional materials^20^, which offers the highest spatial degrees of freedom, suiting fine wavefront engineering and holography^21–23^. However, with array scaling, the control and power distribution networks become bulky, and power and timing pressure escalate rapidly. The second is aperture uniform driving, in which a common stimulus modifies the effective conductivity state across the entire aperture, yielding global amplitude or phase changes^24–26^. While wiring and packaging are simplified, the control dimensionality is limited, typically supporting only binary or low-order modulation (e.g., OOK), making it difficult to directly realize multilevel or higher-order formats. In terms of modulation rate and supported formats, the dynamic bandwidth of electronically active metasurface modulators is generally constrained to the tens of MHz or few GHz^27,28^.

In recent years, demonstrations based on HEMT hybrid metasurface have begun to report continuous electronic modulation, opening the door to higher-order formats and front-end logic decisions^29–31^. To address the trade-off between the degrees of freedom and engineering complexity, we propose and experimentally realize a multi-subarray programmable THz metasurface. Leveraging the high-speed depletion capability of AlGaN/GaN HEMTs, the platform supports amplitude-dominant multidimensional coding. This subarray-level paradigm combines the strengths of both existing approaches: it simplifies wiring and timing complexity relative to unit-cell addressing, while retaining a space combinational state to realize high-order amplitude modulation and optical logic.

**Fig. 1:**
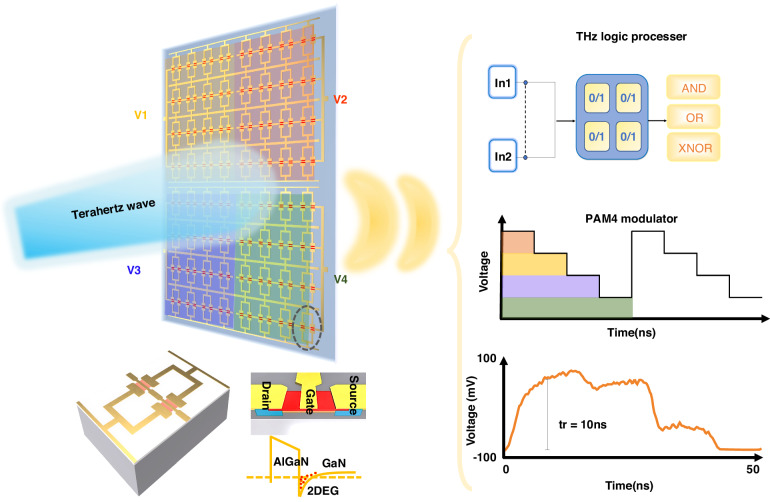
Subarray gated programmable THz metasurface for optical logic and PAM-4. Device concept: four independently gated subarrays composed of periodically repeated AlGaN/GaN-HEMT meta-atoms. Inset: meta-atom layout and band diagram illustrating the gate-controlled 2DEG

As shown in Fig. 1, the metasurface consists of four independently gated subarrays (V1–V4), each formed by a periodic array of HEMT-integrated meta-atoms. Biasing a given subarray therefore tunes the subarray-scale collective resonance, enabling broadband (170–260 GHz) transmission control. All subarrays share a common ground, while their gates are routed by a distributed network to external bias sources. For optical logic, any pair of subarrays is treated as binary inputs; mapping high transmission to “1” and low transmission to “0”, Boolean functions (AND, OR, XNOR) are realized by appropriate bias combinations and a fixed decision threshold on the measured transmission. For multilevel signaling, the four subarrays are partitioned into two groups, {V1, V2} and {V3, V4}, driven by two independent square-wave channels, each split to two subarrays. The amplitude contributions of the two groups add to yield four well-separated transmission levels, implementing PAM-4 at the wavefront level. A representative 220-GHz time trace at 20 MHz shows a clean four-level waveform with a rise time of ~10 ns.

## Results

### Design concept and working principle

**Fig. 2:**
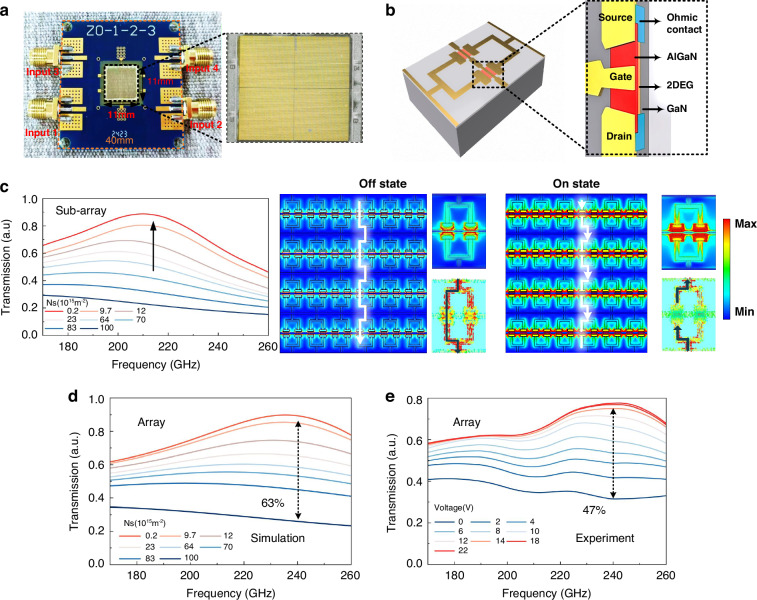
Design and broadband modulation characteristics of the device. **a** Photograph of the fabricated device and detailed microscopic view of the AlGaN/GaN HEMT-based chip. The black dashed outline indicates the chip size 11 × 11 mm, and the orange dashed outline indicates the overall device size 40 × 40 mm. **b** Illustration of a single meta-atom structure highlighting the integrated depletion-mode AlGaN/GaN HEMTs. **c** Simulated transmission spectra demonstrating broadband amplitude modulation capability (170–260 GHz) achieved by switching between OFF and ON states. Simulated electric field distributions demonstrate the distinct modulation mechanism under OFF state (high carrier density, Ns = 10¹³ cm⁻²) and ON state (low carrier density, Ns = 10¹¹ cm⁻²). Meta-atom simulation results are provided in the Supplementary Note 2. **d**, **e** Simulated and measured full-array transmission under gate-voltage sweeps

Figure 2 illustrates the fabricated terahertz modulation chip and its packaging. The device employs a 150-μm SiC substrate, where a metallic microstructure is monolithically integrated with an AlGaN/GaN heterostructure using standard semiconductor processing. The chip comprises four independently addressable subarrays, each comprising 80 × 60 HEMT meta-atoms. In each meta-atom, a pair of depletion mode AlGaN/GaN HEMTs is symmetrically embedded at the gap of a rectangular ring, as shown in Fig. 2b. Owing to spontaneous and piezoelectric polarization at the AlGaN/GaN interface, a high-density 2DEG channel forms at zero bias, with intrinsic carrier mobility > 2200 cm² V⁻¹ s⁻¹ and sheet density > 10¹³ cm⁻². The terminals of the ring are connected via ohmic contacts to the HEMT source and drain and tied to a common ground return. The Schottky gate is routed along the center line of the heterointerface; the gate lines of the four subarrays are fully independent, while a shared ground network ensures a common reference.

When a gate bias is applied, the electric field across the Schottky barrier modulates the 2DEG sheet density, affording broadband tunability of THz transmission. As shown by the subarray-level simulations in Fig. 2c, controllable transmittance is obtained over 170–260 GHz. Under gate control, the sheet density can be tuned from approximately 1 × 10¹⁷ m⁻² down to 0.2 × 10¹⁵ m⁻², driving a transition from the OFF state (low transmission, high 2DEG density) to the ON state (high transmission, depleted 2DEG). In the OFF state, at the meta-atom level the high carrier density preserves a continuous 2DEG current path bridging the split ring; at the subarray level, adjacent meta-atoms couple to form a long-dipole current channels and a subarray-scale collective resonance, which suppresses transmission over a broad band. In the ON state, carrier depletion interrupts the current path and breaks the collective resonance; the resonance becomes localized within each meta-atom, with fields concentrated near the ring edges, as shown in the field maps of Fig. 2c.

Figures 2d, e show the full-array simulations and measurements. Gate voltage sweeps confirm continuous transmission tuning across 170–260 GHz; at 0.24 THz, the transmittance increases from 30% to 78% with gate bias, yielding a 47% modulation depth and a saturation voltage of 14 V. The measured depth is lower than the simulated value due to parasitic elements.

**Fig. 3:**
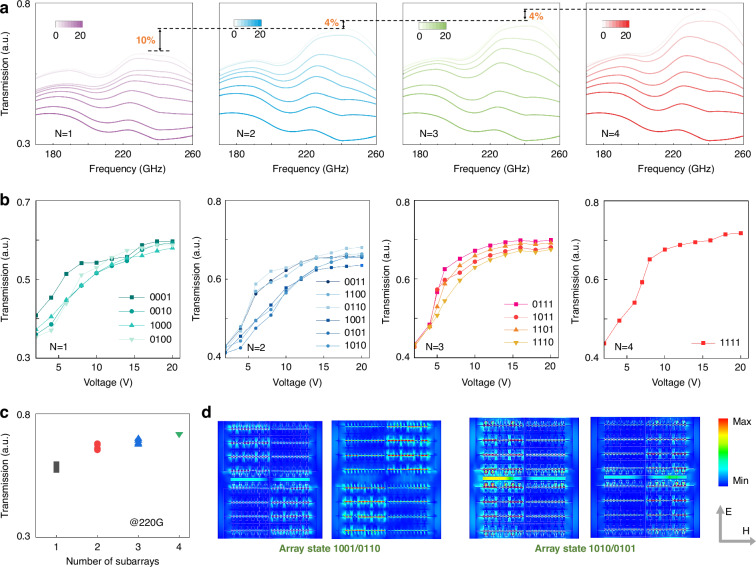
Subarray combination characteristics and transmission modulation. **a** Broadband transmission spectra under different subarray activation patterns, from 1 active subarray (0001) to full activation (1111). As the number of active subarrays increases, the modulation depth decreases but the maximum transmission increases, forming a monotonic yet saturating amplitude envelope. **b** Voltage-dependent transmission curves at 220 GHz for all 16 subarray activation codes, grouped by the number of active subarrays (*N* = 1, 2, 3, and 4). Clear differences exist among single-subarray cases, while multi-subarray combinations tend to converge. **c** Statistical transmission levels versus the number of active subarrays. The average transmission increases, while the variation among combinations at each level becomes smaller. **d** Simulated electric field intensity maps at 220 GHz for array state 1001/0110 and 1010/0101

By applying selected gate voltage combinations across the four subarrays, we selectively modulate the metasurface’s collective resonances, thereby generating discrete THz transmission amplitude states. The independent subarray-level gate-coding scheme is adopted, where bit “1” denotes application of a bias *V*_*H*_ (high transmission) and bit “0” denotes a bias *V*_*L*_ (low transmission). Figure 3a reports transmission spectra over 170–260 GHz as the number of active subarrays varies from *N* = 1 to *N* = 4; for each *N*, gate voltages are swept over *Vg* = 0–20 V. Increasing *N* raises transmission over the entire band until it saturates. At 240 GHz, the corresponding modulation depths are approximately 30%/40%/44%/47% for *N* = 1/2/3/4, respectively.

Fixed frequency measurements at 220 GHz across all 16 binary codes yield the transmission versus voltage characteristics in Fig. 3b, while Fig. 3c summarizes the 220 GHz transmission levels for the number of active subarrays. The data indicate that the number of activated subarrays is the dominant the transmission response, whereas the specific binary arrangement plays a secondary role. Increasing *N* introduces more active control elements and strengthens the THz–2DEG interaction; however, the transmission does not scale linearly with *N*. The observed nonlinear saturation is attributed to collective coupling when multiple subarrays are simultaneously biased, meaning that the subarray collective resonance evolves from localized modes to in-plane cooperative modes (see Supplementary Note 4).

In Fig. 3b, the four *N* = 1 curves (activating each subarray individually) nearly coincide, indicating fabrication uniformity. For *N* = 2, the pairs 0011/1100 (horizontal neighbors) and 1010/0101 (vertical neighbors) are nearly overlapping, whereas the diagonal combinations 1001 and 0110 split under bias. This implies that the device geometry and bias network preserve horizontal/vertical mirror symmetry but lack strict C4 rotational symmetry, rendering the two diagonal states inequivalent. The corresponding field maps in Fig. 3d corroborate this interpretation: for 1001/0110, the coupling channels across the OFF-subarray boundaries differ in location and strength, while 1010/0101 exhibit mirror symmetric field patterns. For *N* = 3, the transmission becomes more stable, and the marginal gain diminishes rapidly. As more subarrays are activated, spatial electromagnetic coupling field amplitude saturates, and the response becomes less sensitive to variations among individual subarrays, resulting in a more uniform and robust combined behavior.

### Optical logic with subarray programmable THz metasurfaces

**Fig. 4:**
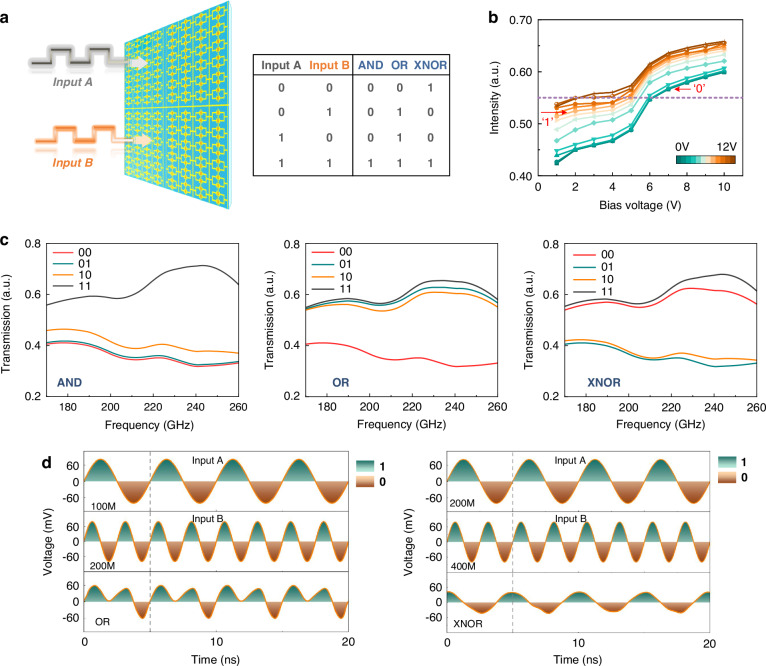
Programmable optical Boolean operations. **a** Schematic of optical Boolean computation using two subarrays (A and B) as logic inputs, and the truth table for AND/OR/XNOR is shown. **b** Transfer characteristics at 220 GHz: output intensity versus selector-subarray bias. Colored curves denote different gate voltages applied to the input subarrays; the dashed horizontal line indicates the decision threshold (Tth≈0.55) separating True and False. Bias tuning enables switching of the logical outcome for the same input pattern. **c** Measured transmission spectra from 170 to 260 GHz for the four input combinations under three logic functions (AND, OR, XNOR). **d** Left: OR logic with 100 MHz and 200 MHz inputs. Right: XNOR logic with 200 MHz and 400 MHz inputs. The top two traces are the input voltages; the bottom trace is the logic output after thresholding. Shaded regions denote digital “1/0”

Exploiting the coding flexibility of subarray programmable THz metasurfaces, AND, OR, and XNOR logic operations can be realized concurrently. As shown in Fig. 4a, two independently fed subarrays (denoted A and B) serve as the logical inputs; their ON/OFF combinations constitute a 2-bit input set [00,01,10,11]. Gate conditions of *V* = 0 V and *V* = 12 V define logical “0” and “1”, respectively. The output is read from the measured transmittance: high transmittance corresponds to True, and low transmittance to False. The remaining two subarrays act as DC-biased selector subarrays that choose the logic function. Figure 4b plots the logical subarray output as the selector subarray bias is varied: the abscissa represents the bias, and the colored curves indicate the logical subarray output for different gate voltages under input states. By tuning the selector bias, the transmittance levels associated with 00/01/10 states can be linearly adjusted. For illustration, with a decision threshold *T*_th_ = 0.55, logic “0” and “1” may be assigned to input gates of 0 V and 10 V, respectively; under selector bias control, the 01/10 cases can yield either True or False, enabling logic gate programmability. As shown in Fig.4c, the device implements broadband Boolean operations (AND, OR, and XNOR) over 170–260 GHz. Benefiting from array level 2DEG tunability, the metasurface maintains stable, discriminable logic outputs across a wide band, in contrast to prior devices limited to narrowband or single-frequency logic. A frequency dependent performance factor *P(f)* for the three logic gates (see Supplementary Note 5) exceeds the threshold over 200–260 GHz, confirming broadband logical robustness.

The ultrafast response of the logic operations is further validated in Fig. 4d. Two RF sources, synchronized to a common clock and provided with suitable DC offsets, generate sinusoidal inputs, Input A and Input B, that are fed to the two logical subarrays. Positive half-cycles encode logic “1”, and negative half-cycles encode logic “0”. In the left panel of Fig. 4d, Input A = 100 MHz and Input B = 200 MHz; within one period, the effective input coding sequences correspond to 1100 and 1010, and the measured output is 1110, implementing OR logic at 100 MHz. In the right panel, Input A = 200 MHz and Input B = 400 MHz; the output sequence 1001 realizes XNOR logic at 200 MHz. These results demonstrate that the subarray coding platform supports programmable and dynamic Boolean operations.

### Real-time quasi optical link and PAM-4 modulation

**Fig. 5:**
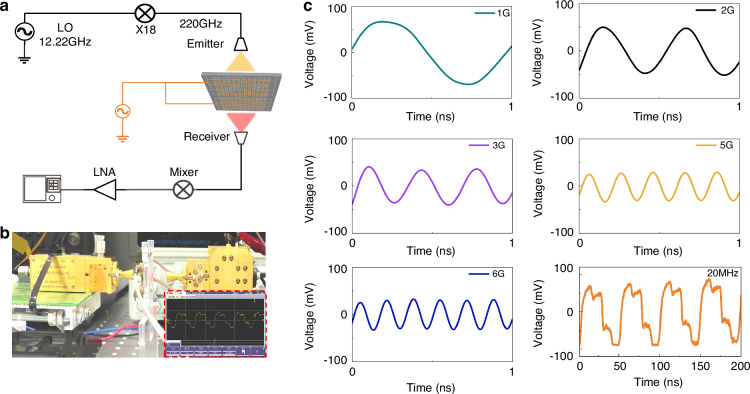
220-GHz quasi-optical link and real-time modulation measurements. **a** Block diagram of the testbed. **b** Photograph of the experimental setup; inset shows a representative waveform captured during measurements. **c** Time-domain traces demonstrating single-tone tracking at 1, 2, 3, 5, and 6 GHz and a PAM-4 waveform at 20 MHz (right/bottom panel; 200 ns window), exhibiting four clearly resolved amplitude levels

The digitally addressable subarray architecture affords finely quantized control of transmission amplitude, providing the physical basis for high-order modulation. To evaluate link level performance in a communications setting, a 220 GHz quasi optical RF testbed was constructed (Fig. 5a). A 12.22 GHz local oscillator was ×18 frequency-multiplied to generate the 220 GHz carrier, which was radiated by a horn toward the metasurface under test. Modulation waveforms from an arbitrary waveform generator (AWG) were applied to the gate network via a bias-tee superimposed on the DC bias, thereby imprinting amplitude modulation on the transmitted field. On the receive side, the signal collected by a horn is down converted in a mixer, amplified by a low noise amplifier (LNA), and recorded as an intermediate frequency waveform on a real-time oscilloscope. Figure 5b shows the setup and representative traces, while Fig. 5c shows the single tone response supports up to 6 GHz modulation bandwidth.

Real-time high-order modulation is further demonstrated using two synchronized function generators that produce 20 MHz and 40 MHz square waves. Each waveform is power split to drive two subarrays, enabling synchronous control of all four subarrays. The received signal exhibits four distinct amplitude levels, confirming successful generation and detection of PAM-4, corresponding to a theoretical link bandwidth of 80 MHz, as shown in Fig. 5c.

Because the four subarrays switch concurrently, inter-subarray coupling is enhanced, and the effective RC time constant of the distributed gate network increases. These effects soften the edges (rise/fall time 10 ns) and introduce mild clipping in the measured PAM-4 waveform. Even with some nonuniform level isolation, the metasurface delivers stable multilevel discrimination, demonstrating practical usability and engineering promise in link-level THz communications.

## Discussion

This work proposes and experimentally validates a subarray gate-controlled, programmable THz metasurface. The architecture elevates the subarray to the minimum controllable unit: combinatorial subarray coding enables dynamic optical Boolean logic, while two independent gate drives applied to two subarray groups produce a weighted superposition that realizes four-level PAM-4 with direct wavefront mapping. AlGaN/GaN HEMTs serve as the active tunable material; for representative geometry and material parameters, the intrinsic RC ceiling is on the order of ~30 GHz (see Supplementary Note 6). The measured single tone modulation bandwidth of 6 GHz indicates that packaging and bias network parasitics constitute the dominant present constraint.

Scaling from a 2 × 2 to a larger N × N array increases the number of addressable subarrays, and the count of discrete coding states grows exponentially as 2^*N*^. This vast state space suggests significant potential for implementing high-order multilevel mapping and parallel logic operations on a single hardware platform. However, from a practical standpoint, the realizable capacity for multilevel amplitude modulation is not infinite. As the number of activated subarrays increases, stronger array-level coupling induces nonlinear compression of the transmission levels, resulting in non-uniform level spacing and diminishing marginal gains in modulation depth. Consequently, despite the theoretical abundance of coding states, reliable implementation of high-order schemes such as PAM-16 becomes challenging due to level overlap and limited noise tolerance. We anticipate that PAM-8 represents a feasible practical limit for reliable differentiation in the current configuration, with fabrication uniformity and signal-to-noise ratio being the primary parameters limiting further scalability. To push beyond this limit, look-up table linearization or digital predistortion will be essential to counteract nonlinear compression.

Furthermore, advancing this technology requires addressing the intrinsic trade-off between modulation speed and depth. Scaling up the array size introduces stronger inter-subarray coupling and parasitic effects, which can degrade the available bandwidth and induce amplitude uneven. Simultaneously, achieving higher modulation depth typically implies increasing the capacitive load, which restricts modulation speed. Therefore, future work must focus on further optimizing the balance between these conflicting factors. To address these constraints, specific strategies such as optimizing the high-integration structure to reduce parasitic capacitance^32,33^, upgrading from wire-bonding to via-hole interconnects to minimize inductance, and employing digital predistortion are critical for maintaining signal integrity. Future directions include heterogeneous integration and emerging tunable materials to further relax scaling bottlenecks. Ultimately, balancing these device optimizations with system integration strategies will be key to unlocking the full potential of programmable THz metasurfaces for high-speed, multi-level communication systems.

## Methods

### Sample fabrication

The device was fabricated on commercial GaN-on-sapphire wafers using standard photolithographic processes. First, alignment marks were defined by photolithography, magnetron sputtering, and lift-off to ensure high precision mask registration in subsequent steps. The active regions were then isolated by ion implantation to suppress leakage. The multilayer metal stacks were deposited on the source/drain regions and rapid-thermal annealed to form ohmic contacts to the 2DEG channel. The metasurface pattern was subsequently defined by photolithography followed by electron beam evaporation of Ni/Au layer and lift-off on the GaN layer. Additional Ni/Au metal was deposited on the control electrode pads to increase thickness and improve wire-bond reliability. For substrate thinning, the wafer was temporarily bonded, mechanically lapped and polished to the target thickness, and then diced. An individual chip was bonded onto PCB with a two-component epoxy adhesive, and the control electrodes were wire-bonded to the external driving circuitry.

### Numerical simulations

Full-wave electromagnetic simulations were performed using the commercial simulation software CST microwave studio. In the simulation, a single meta-atom of the metasurface structure is simulated with periodic boundary conditions employed in axial directions orthogonal to the incident waves; for a full-array model, open (add space) boundary conditions were used on all sides. GaN-HEMT material characteristics are described by a Drude model (see Supplementary Note 7).

### THz measurements

The experiments comprise S-parameter measurements, dynamic optical logic, and PAM-4 modulation. First, the metasurface is characterized with a vector network analyzer in a free space setup using two horn antennas and 170–260 GHz T/R modules. A THz response through a bare substrate is recorded and used as a reference to normalize the device response. Then, the integrated platform is constructed for ultrafast dynamic optical logic, and PAM-4 modulation, the frequency-multiplied RF chain shown in Fig. 5a is employed. An AWG (Keysight M8194A) provides the gate-drive waveforms for the metasurface, and the received waveform is recorded in real time by a digital oscilloscope operating at 5 GSa/s.

## Supplementary information

Supplementary Information of Manuscript

## Data Availability

The authors declare that all relevant data are available in the paper and its Supplementary Information Files, or from the corresponding author on request.
