# Supplementary material for: Subarray programmable terahertz metasurface for optical logic and high-order amplitude modulation
Source: Light Sci Appl. 2026 May 7;15:222. doi: 10.1038/s41377-026-02255-z (PMC13153363; doi:10.1038/s41377-026-02255-z)
Supplement: Supplementary file 1 — Supplementary Information of Manuscript [file 41377_2026_2255_MOESM1_ESM.docx]

Supplementary Information for

**Subarray-programmable terahertz metasurface for optical logic and high-order amplitude modulation**

Lan Wang^1^*, Sen Gong^2,3^, Chaoming Xia^2^, Dongyang Liu^1^, Xuan Cong^2^, Ao Zhu^2^, Hongxin Zeng^2,3^, Feng Lan^2,3^, Ziqiang Yang^2,3^, Taiichi Otsuji^4,5*^, and Yaxin Zhang^2,3^*

^1^School of Physics, University of Electronic Science and Technology of China, Chengdu 611731, China.

^2^Engineering Center of Integrated Optoelectronic & Radio Meta-chips, Chengdu 611731, China.

^3^School of electronic Science and Engineering, University of Electronic Science and Technology of China, Chengdu 611731, China.

^4^Center of Excellence ENSEMBLE3 Ltd., Warszawa 01919, Poland.

^5^International Research Institute of Disaster Science, Sendai 9808572, Japan.

*email: [wanglan@uestc.edu.cn](mailto:wanglan@uestc.edu.cn), [taiichi.otsuji@ensemble3.eu](mailto:taiichi.otsuji@ensemble3.eu), [zhangyaxin@uestc.edu.cn](mailto:zhangyaxin@uestc.edu.cn).

**Supplementary Note 1: Comparison of electrically controlled THz metasurfaces**

Table 1 surveys representative electrically driven THz metasurfaces. Fine per-pixel control, typically implemented in CMOS technology, enables beam steering and holography. However, the high I/O count and dense clock distribution make large aperture scaling at THz difficult and constrain further symbol rate gains ^[1-2]^. Aperture-level control metasurface reported electric modulation speeds from tens of MHz or few GHz, but their addressing scheme limits analog front-end flexibility; moreover, approaches that rely on unit resonance frequency shifts can deliver large modulation depth at the cost of narrow operating broadband range ^[3-4]^. Subarray driving strategies (or super-unit cell) have been explored to simplify control complexity ^[5-7]^. However, these works are often demonstrated at microwave frequencies using light-controlled biasing or slow thermal mechanisms, resulting in quasi-static reconfigurability rather than the high speed modulation required for real-time THz communications.

This work adopts the independently addressable subarray as the minimum controllable unit based on HEMT, striking a balance between control complexity and functional density, expanding the discrete state space with modest I/O growth. Unlike prior quasi-static microwave or optical approaches, our design leverages collective resonance switching to enable GHz modulation speeds. It unifies front-end functionality, delivering ultrafast Boolean logic and PAM-4 modulation with link-level validation in a 220 GHz quasi-optical system.

**Table 1** Comparison of terahertz metasurface technologies using electrical control

|  | **Technology** | **Addressable granularity** | **Control parameter** | **Functionality** | **Response speed** | **Broadband range** | **Ref.** |
| --- | --- | --- | --- | --- | --- | --- | --- |
| 1 | 45nm CMOS | Unit cell | Phase | Beam  steering |  | 0.37-0.41 THz | [1] |
| 2 | 65nm CMOS | Unit cell | Phase and binary-amplitude | Beam  steering and holographic | 5 GHz | 0.3 THz | [2] |
| 3 | GaN HEMT | Aperture | Amplitude | Optical logic | 3 GHz | 0.286 THz | [3] |
| 4 | Graphene-based metasurface | Aperture | Amplitude | Amplitude modulation | 30 MHz | 2.15 THz | [4] |
| 5 | VO_2_-based metasurface | Subarray | Phase | Beam  steering | 24 us | 0.425 THz | [5] |
| 6 | GaN SBD | Subarray | Phase | Beam  steering | 200 MHz | 0.319 THz | [6] |
| 7 | Varactor | Super-unit cell | Phase | Beam  steering |  | 3.75/4.02 GHz | [7] |
| **This work** | GaN HEMT | Subarray | PAM-4 amplitude | Optical logic and PAM-4 modulation | 6 GHz | 0.17-0.26 THz |  |

**Supplementary Note 2: Meta-atom design and simulation performances**


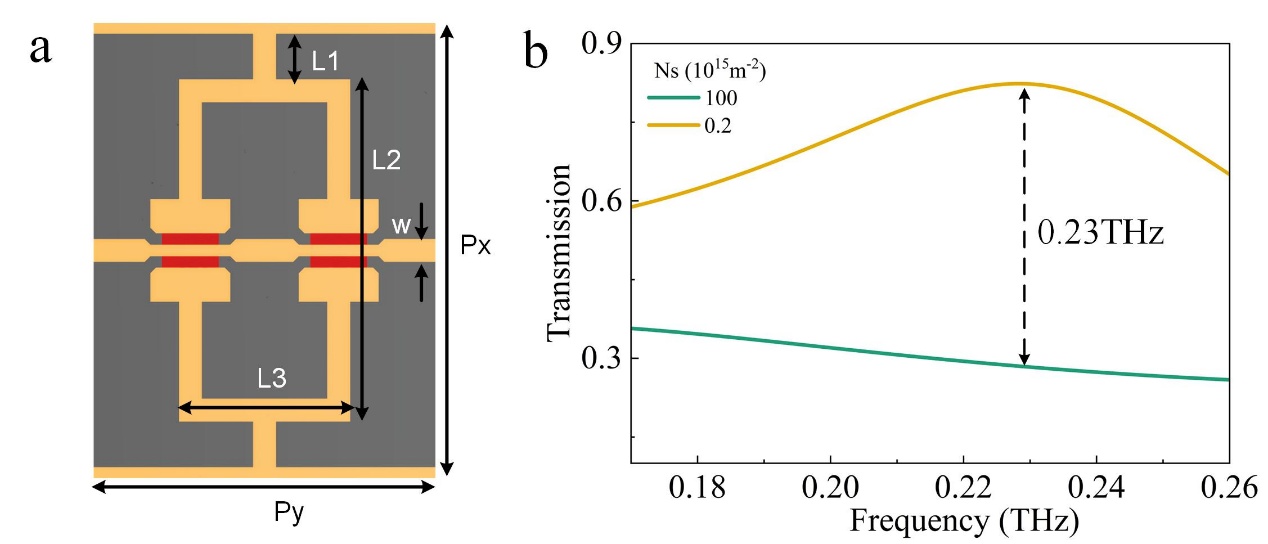


**Fig. S1** Unit-cell geometry and simulation results.

The device uses a **150 µm** SiC substrate; metal linewidth *w*=4 μm; lattice periods *P*x=50 μm and *Py*=90 μm; and rectangular-ring dimensions *L*1=8 μm, *L*2=60 μm, and *L*3=30 μm. Full wave simulations were performed in CST Studio Suite. For the unit cell model, unit-cell boundary conditions with Floquet ports were used; the resulting resonance frequency agrees closely with the 0.24 THz resonance observed in full array simulations. The subarray model employed finite aperture electromagnetic boundaries, a magnetic boundary along the polarization direction and an electric boundary orthogonal to it, to compute the subarray transmission. The smaller effective aperture and different boundary conditions in the subarray configuration account for the slight resonance shift relative to the full array case.

**Supplementary Note 3: Simulation results of inter-subarray spacing**

The impact of inter-subarray spacing *d* that defined as the vertical distance between adjacent subarrays along the polarization direction of the incident electric field is illustrated in Fig. S2. Fig.S2a and b show the full array field distributions at *d*=10 µm and *d*=40 µm under both ON and OFF states. Strong capacitive coupling arises between neighboring subarrays at *d*=10 µm, leading to mutual interference. Conversely, larger spacings elevate the transmission floor, thereby compressing the achievable modulation depth, as confirmed by the results in Fig. S2c.


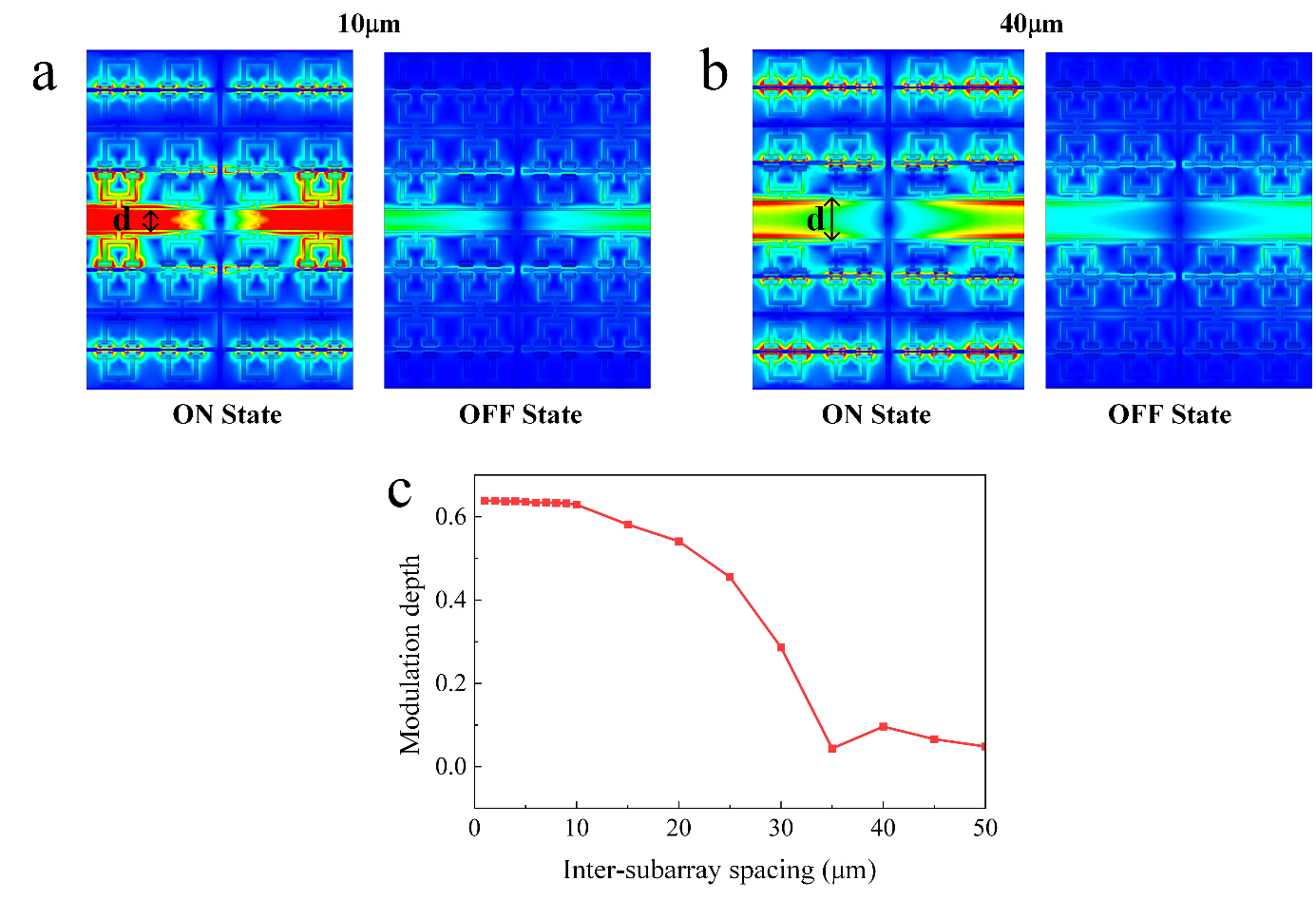


**Fig. S2** Simulation results of inter-subarray spacing. **a**, **b**. Simulated electric field distributions of the metasurface under the ON state (short dipole moment) and OFF state (long dipole moment) for inter-subarray spacings of 10 µm and 40 µm, respectively. Strong parasitic coupling fields are observed between adjacent subarray feedlines, which degrade the flatness of higher-order modulation. **c**. Dependence of modulation depth on the inter-subarray spacing.

**Supplementary Note 4: Statistical uniformity of subarray coding transmittance**

As shown in Fig.S3a, when a single subarray is activated, the field remains localized within the array; as the number of active subarrays increases, the electromagnetic coupling evolves from localized modes toward array-level collective resonances, leading to field saturation. Fig.S3b shows transmittance distribution at 240 GHz for all 16 coding states. The statistical standard deviation of transmittance across all coding combinations is summarized in Fig.S3c, where the solid line denotes the fitted curve using the following relation:

$$\sigma\left( T \right)=\sigma_{0}e^{-k\left( N-1 \right)}$$

where *N* denotes the number of activated subarrays. The exponential decay of σ(*N*) indicates that, as more subarrays are activated, the variability among different coding combinations is suppressed, and transmittance is primarily determined by the number of active subarrays rather than the specific coding pattern.


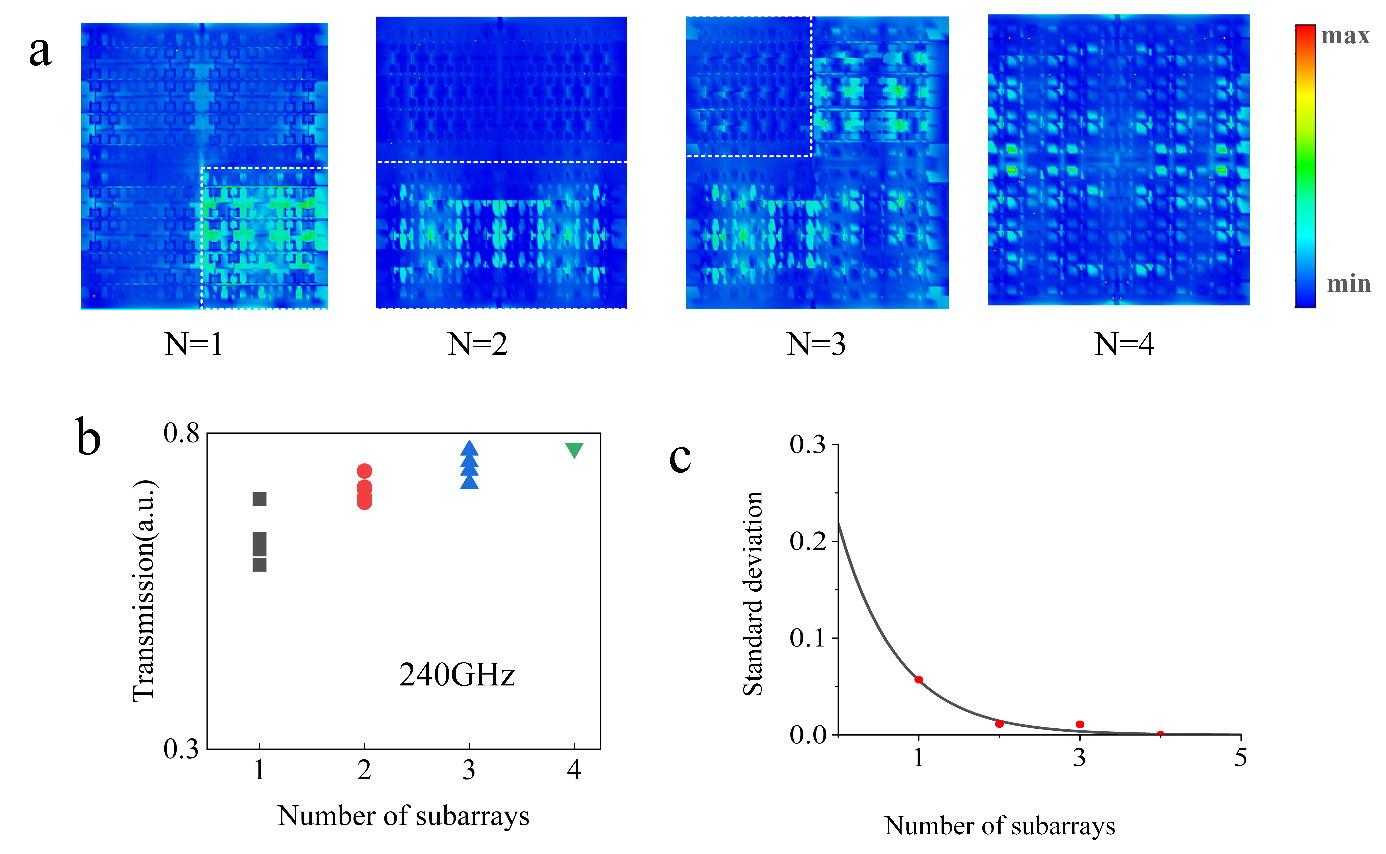


**Fig. S3** Subarray activation and transmittance statistic. **a**. Simulated electric field distributions under different numbers of activated subarrays. **b**. Transmittance distribution at 240 GHz for all 16 coding states. **c**. Standard deviation of transmittance among different coding combinations

**Supplementary Note 5: Performance factor of logic functions**


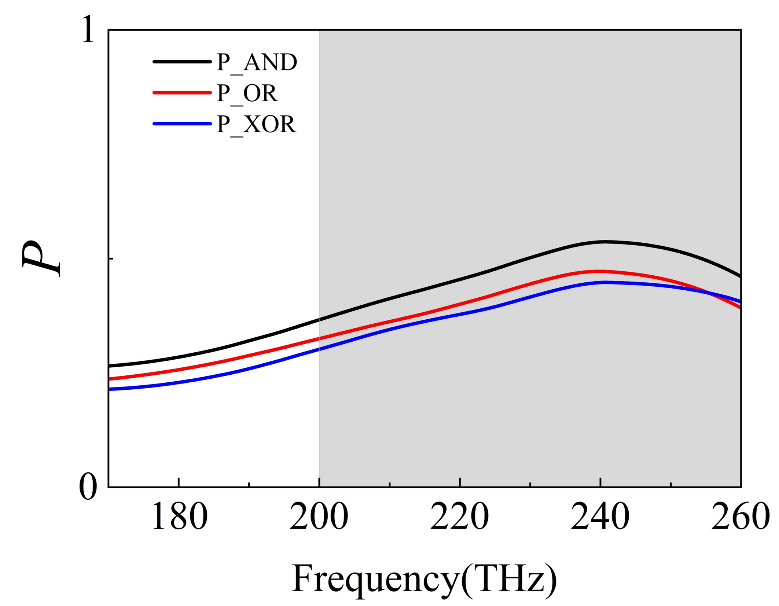


**Fig. S4** The performance factors of the logic gates

To more accurately assess the logic operation capability of the device across different frequencies, we introduce a logic performance factor P(*f*)：
$$P\left( f \right)=\frac{H\left( f \right)-L\left( f \right)}{H\left( f \right)+L\left( f \right)}-\sigma_{H}\left( f \right)-\sigma_{L}\left( f \right)$$

$$\sigma_{H}\left( f \right)=\sqrt{\frac{1}{m}\sum_{i=1}^{m} \left( T^{i}\left( f \right)-H\left( f \right) \right)^{2}}$$

$$\sigma_{L}\left( f \right)=\sqrt{\frac{1}{n}\sum_{i=1}^{n} \left( T^{i}\left( f \right)-L\left( f \right) \right)^{2}}$$

where H(f) and L(f) represent the average transmission levels of all logic states corresponding to outputs “1” and “0”, respectively, at a given frequency *f*. The terms $\sigma_{L}\left( f \right)$ and $\sigma_{H}\left( f \right)$ denote the standard deviations within each group. This metric simultaneously accounts for both inter-state distinguishability and intra-group consistency. A higher *P* value indicates clearer logic discrimination and more stable output performance. Generally, when P>0.3 the logic states are considered to exhibit sufficient distinguishability and stability to support robust logic operation. Conversely, frequency intervals with P<0.1 are deemed unreliable for logic interpretation due to poor contrast or inconsistent outputs. The results show that in the 200–260 GHz range, all three types of logic gates, under various sub-array combinations, yield P-values consistently above the defined threshold. This confirms the broadband stability of the logic operation enabled by the metasurface platform.

**Supplementary Note 6: Intrinsic response time of HEMT meta-atom**

To estimate the ultimate dynamic response of the metasurface modulator, the intrinsic RC time constant of each HEMT unit cell was evaluated, as this fundamentally constrains the maximum achievable modulation speed. The time constant is defined as

$$\tau=R_{channel}\times C_{gate}$$

where R_channel_ is the resistance of the 2DEG channel, and C_gate_ is the capacitance between the gate electrode and the 2DEG layer. The channel resistance can be approximated as

$$R_{channel}=\frac{L}{q\mu n_{2D}W}$$

with *L* and *W* denoting the gate length and width, respectively; *q* the elementary charge; *μ* the electron mobility; and *n*_2D_ the sheet carrier density of the 2DEG. The gate capacitance is modeled as:

$$C_{gate}=\frac{\varepsilon_{r}\varepsilon_{0}WL}{d}$$

where *ε_r_* is the relative dielectric constant of the AlGaN barrier, and *d* is the barrier thickness. Thus, the relationship indicates that the modulation speed is primarily governed by the gate length *L*, barrier thickness *d*, and material parameters *μ* and *n*_2D_ ​. For representative values (*L*=2 μm, *n*_2D_=10^13^ cm^−2^, μ=1500 cm^2^/V·s, d=25 nm, *ε*_r_=9), the RC time constant is estimated to be ~4 ps, corresponding to a modulation bandwidth exceeding 30 GHz.

**Supplementary Note 7: Drude model of AlGaN/GaN HEMT**

Fig. S5 shows a cross-section diagram of the AlGaN**/**GaN HEMT structure, including key parameters of the device ^[8]^. In the CST simulation, we adopted the Drude model to characterize the dynamic transport properties of the 2DEG ^[9]^. The equivalent complex permittivity of the 2DEG is expressed as:

$$\text{ε}\text{(}\text{ω}\text{)=}\text{ε}_{\text{∞}}\text{+}\text{j}\text{ω}_{\text{p}}^{\text{2}}\frac{\text{γ}\text{ω}^{\text{-1}}}{\text{ω}^{\text{2}}\text{+}\text{γ}^{\text{2}}}$$

where $\text{ε}_{\text{∞}}$ denotes the real part of the complex permittivity $\text{ε}_{\text{∞}}\text{=}\text{ }\text{9.8}\text{ε}_{\text{0}}$, i.e., the permittivity of GaN and $\text{ε}_{\text{0}}$ is the vacuum permittivity. In the imaginary part, $\text{ω}_{\text{p}}$ represents the plasma angular frequency, which is intrinsically dependent on the 2DEG carrier density $\text{N}_{\text{s}}$, $\text{ω}$ denotes the angular frequency of the incident wave; and $\text{γ}$ stands for the electron collision frequency.

**Fig. S5** Section diagram of HEMT region.

To ensure simulation accuracy, the 2DEG layer is divided into one central gated region and two adjacent non-gated regions. Specifically, the electron sheet density in the non-gated region is maintained at its initial value of 1×10¹⁷ m⁻², while the concentration in the gated region is modulated by the bias voltage, ranging down to a depletion minimum of 0.2×10¹⁵ m⁻². To account for the fringing electrostatic field effects observed in practical devices, the effective length of the gated simulation region is set to 2.3 μm, extending the physical gate length 2 μm. Additionally, to model the equivalent ohmic contact resistance, a dielectric layer (thickness: 20 nm, resistivity: 412 Ω·μm) is inserted between the source and drain electrodes and the 2DEG layer.

**References**

1. Yang, Y., Gurbuz, O. D. & Rebeiz, G. M. An Eight-Element 370–410-GHz Phased-Array Transmitter in 45-nm CMOS SOI With Peak EIRP of 8–8.5 dBm. *IEEE Trans. Microw. Theory Tech.* **64**, 4241–4249 (2016).

2. Venkatesh, S., Lu, X., Saeidi, H. & Sengupta, K. A high-speed programmable and scalable terahertz holographic metasurface based on tiled CMOS chips. *Nat. Electron.* **3**, 785–793 (2020).

3. Zeng, H. *et al.* Dynamically logical modulation for THz wave within a dual gate–controlled 2DEG metasurface. *Sci. Adv.* **10**, eadr1448 (2024).

4. Xia, R. *et al.* Achieving 100% amplitude modulation depth in the terahertz range with graphene-based tuneable capacitance metamaterials. *Light Sci. Appl.* **14**, 256 (2025).

5. Chen, B. *et al.* Electrically addressable integrated intelligent terahertz metasurface. *Sci. Adv.* **8**, eadd1296 (2022).

6. Yu, R. *et al.* A GaN Schottky Barrier Diode-Based Terahertz Metasurface for High-Precision Phase Control and High-Speed Beam Scanning. *Adv. Mater.* 2507534 (2025).

7. Zhang, G. *et al*. Light‐controllable digital coding metasurfaces. *Adv. Sci*. **5**, 1801028 (2018).

8. Zeng, H. *et al*. Dynamically logical modulation for THz wave within a dual gate–controlled 2DEG metasurface. *Sci. Adv*. 10, eadr1448 (2024).

9. Shrekenhamer D. *et al*. High speed terahertz modulation from

metamaterials with embedded high electron mobility transistors. O*ptics express*, **19**, 9968-9975 (2011).
